# Synthesis, characterization, and adsorptive removal of methyl blue using combustion-synthesized Al_4_B_2_O_9_ and Al_5_BO_9_

**DOI:** 10.1039/d6ra01929c

**Published:** 2026-06-04

**Authors:** Uğur Özkan, Okan Bayram, Emel Moral, İlhan Pekgözlü, Fethiye Göde

**Affiliations:** a Department of Forest Products Engineering, Faculty of Forestry, Isparta University of Applied Sciences 32200 Isparta Turkey ugurozkan@isparta.edu.tr; b Department of Chemistry, Faculty of Engineering and Natural Sciences, Süleyman Demirel University 32200 Isparta Turkey okanbayram@sdu.edu.tr; c Department of Chemistry, Graduate School of Natural and Applied Sciences, Süleyman Demirel University 32200 Isparta Turkey emel32moral@gmail.com; d Department of Environmental Engineering, Faculty of Engineering, Karabük University 78050 Karabük Turkey ilhanpekgozlu@karabuk.edu.tr; e Department of Chemistry, Faculty of Engineering and Natural Sciences, Süleyman Demirel University 32200 Isparta Turkey fethiyegode@sdu.edu.tr

## Abstract

This study investigates the removal process of methyl blue (MeB), an anionic dye, using synthesized Al_4_B_2_O_9_ and Al_5_BO_9_ as adsorbent materials. Al_4_B_2_O_9_ and Al_5_BO_9_ were synthesized *via* the combustion method. The structural, morphological, textural, thermal and surface–charge properties of the synthesized borate materials were evaluated using FTIR, XRD, SEM, BET, TGA, and zeta potential measurements. The adsorption behavior of Al_4_B_2_O_9_ and Al_5_BO_9_ was assessed by varying solution pH, contact duration, initial MeB concentration, adsorbent loading, and temperature. Kinetic, equilibrium, and thermodynamic analyses were carried out to clarify the adsorption behavior and possible interaction pathways between MeB and the synthesized borate surfaces. Kinetic modeling showed that the PSO equation most accurately describes MeB removal by both borate materials, as indicated by the high *R*^2^ values. The thermodynamic parameters showed that MeB adsorption was favored by increasing temperature and proceeded spontaneously, as confirmed by the positive Δ*H*° and negative Δ*G*° values, respectively (Al_4_B_2_O_9_: Δ*H* = 16.827 kJ mol; Al_5_BO_9_: Δ*H* = 17.130 kJ mol^−1^). The positive Δ*S*° values indicate that the adsorption process was accompanied by increased interfacial disorder during the transfer of MeB molecules from solution to the borate surface (Al_4_B_2_O_9_: Δ*S* = 80.222 J (mol^−1^ K^−1^); Al_5_BO_9_: Δ*S* = 82.940 J (mol^−1^ K^−1^)). Equilibrium modeling indicated that the Freundlich equation more suitably described MeB uptake by both Al_4_B_2_O_9_ and Al_5_BO_9_, suggesting adsorption on heterogeneous surface sites. According to the Freundlich model, the *K*_F_ values were determined as 21.146 for Al_4_B_2_O_9_ and 27.072 for Al_5_BO_9_. Although the Freundlich model provided the best fit, the Langmuir model was used to estimate the maximum monolayer adsorption capacities, which were calculated as 70.547 mg g^−1^ for Al_4_B_2_O_9_ and 105.785 mg g^−1^ for Al_5_BO_9_. Overall, the results demonstrate the potential of combustion-derived Al_4_B_2_O_9_ and Al_5_BO_9_ as inorganic borate adsorbents for removing anionic MeB from aqueous media.

## Abstract 2

This study investigates the removal process of methyl blue (MeB), an anionic dye, using synthesized Al_4_B_2_O_9_ and Al_5_BO_9_ as adsorbent materials. Al_4_B_2_O_9_ and Al_5_BO_9_ were synthesized *via* the combustion method.

## Graphical abstract

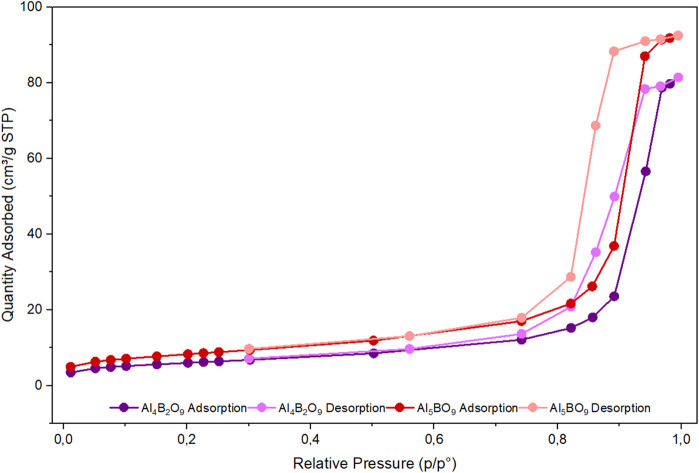

## Introduction

Water resources are essential for ecological balance, public health, and the continuity of agricultural and industrial activities. The growing discharge of chemical and biological pollutants into aquatic environments has made water security a major global concern.^1,2^ Effluents containing synthetic dyes represent a major environmental concern due to their strong coloration, chemical persistence and possible toxic effects. The protection of water resources is essential for sustaining life on Earth and maintaining an environmentally sustainable future.^2,3^ It is evident that industrial and metallurgical wastewater pollutants constitute a significant source of annual water contamination. The dyeing process involves the application of synthetic or natural colorants to materials such as textiles, paper, leather, and plastics in order to impart color.^3,4^ During the discharge of these industrial effluents into surrounding water bodies, substantial amounts of hazardous dyes have been detected. Changes in water quality due to the presence of biological, physical, or chemical components that may pose a potential threat to public health and safety are classified as water pollution. Synthetic dyes constitute an important class of industrial pollutants, particularly due to their extensive use in textile related processes.^1–4^ The discharge of organic dyes into water bodies such as rivers and ponds has been reported to cause severe damage to flora and fauna. This is primarily attributed to the toxic, carcinogenic, and mutagenic nature of these compounds.^5^ Dyes are known to possess complex molecular structures that confer resistance to degradation. Their persistence and strong coloration can limit light transmission in water bodies, which may suppress photosynthesis and decrease dissolved oxygen availability.^6^ Many dyes are toxic substances that may lead to various health problems, including skin irritation, respiratory disorders, and even cancer. Moreover, their complex molecular structures pose significant challenges to natural degradation processes.^7^ Methyl blue (MeB), a compound used in various industries including cotton, silk, and wool dyeing imparts a blue color to the applied material and is commonly detected in industrial wastewater streams. In addition, it has numerous applications in medical, biological, and chemical industries. It has been reported that MeB may cause health complications.^7,8^ MeB is an anionic sulfonated dye that has been frequently used in staining applications, including biological and histological studies. However, it is toxic and biologically harmful to both organisms and the environment.^9,10^ Various physical and chemical processes are employed for water treatment. A range of methods including chemical, biological, and physical approaches are used in the treatment of dye-contaminated wastewater. These include biodegradation, membrane filtration, ion exchange, electrochemical oxidation, and adsorption. Most of these techniques have been extensively investigated and applied.^11^ Among the available treatment strategies, adsorption is widely preferred because it combines practical operation with effective pollutant uptake under different solution conditions.^12^ Therefore, this study evaluates combustion-derived Al_4_B_2_O_9_ and Al_5_BO_9_ as aluminum borate adsorbents for the removal of anionic MeB from water. The adsorption behavior of combustion-synthesized Al_4_B_2_O_9_ and Al_5_BO_9_ toward anionic MeB has not been systematically evaluated in terms of pH, dosage, contact time, temperature, kinetics, isotherms, thermodynamics, and surface charge. Aluminum borates are characterized by favorable stability properties, including high thermal stability, low thermal expansion and conductivity, high creep resistance, and strong corrosion resistance.^13^ Owing to these properties, aluminum borates particularly in the form of nanowires and whiskers are widely utilized in ceramic composites, optoelectronics, and tribological applications.^14^ Therefore, the present study focuses on the synthesis, physicochemical characterization, and adsorption performance of Al_4_B_2_O_9_ and Al_5_BO_9_ toward MeB removal. The synthesized borate phases were examined in terms of crystalline structure, surface functional groups, morphology, textural properties, thermal behavior, and surface charge. Subsequently, adsorption experiments were conducted, and the results were interpreted in terms of fundamental adsorption properties and design parameters to assess the effectiveness of the synthesized materials.

## Materials and methods

### Chemicals and instruments

MeB was selected as the target anionic dye for the adsorption experiments. The properties of the dye, supplied by Isolab (Germany) are presented in Table 1. The chemicals used for synthesis and pH adjustment were purchased from Sigma-Aldrich. The maximum absorption wavelength of MeB was identified as 600 nm by wavelength scanning using Peak Instruments C-7100 and DLAB 1100 UV-Vis spectrophotometers. Solution pH values were measured during the experiments using a Hanna HI2020-02 Edge pH meter. FT-IR spectra were collected with a JASCO FT/IR-4700 Type A spectrometer in the 4000–400 cm^−1^ range to identify the characteristic vibrational features of the synthesized borates. The surface morphology of the synthesized materials was observed using a Quanta-FEG-250 scanning electron microscope and XRD analyses were conducted with a Bruker D8 Advance Twin diffractometer. The surface area and porosity of the Al_4_B_2_O_9_ and Al_5_BO_9_ materials were determined by Brunauer–Emmett–Teller (BET) analysis using a Micromeritics Gemini VII 2390 t instrument. Thermal degradation behavior and mass–loss profiles were investigated by TG/DTG analysis from 25 to 1400 °C. The surface charge behavior of the adsorbents was evaluated using a HORIBA SZ-100 zeta potential analyzer. A Radwag AS220.R2 Plus analytical balance was used to determine solution quantities during the experiments.

**Table 1:** Physicochemical properties of MeB dye

Name	Methyl blue (MeB)
Formula	C_37_H_27_N_3_Na_2_O_9_S_3_
Molecular weight	799.814 g mol^−1^
Type	Anionic
Molecular structure	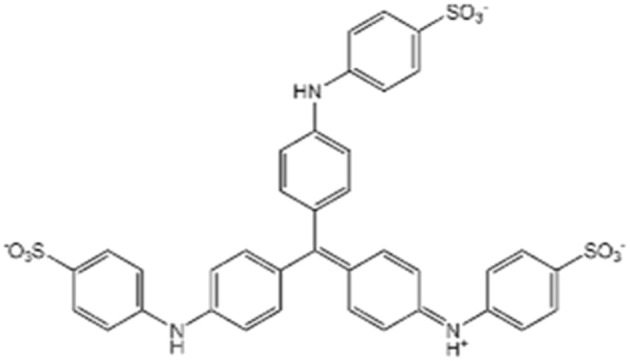

### Synthesis of Al_4_B_2_O_9_ and Al_5_BO_9_

Two aluminum borate materials with the chemical formulas Al_4_B_2_O_9_ and Al_5_BO_9_ were employed in the adsorption experiments. These materials were synthesized using the combustion method. High-purity chemicals supplied by Sigma-Aldrich were used in the preparation of the adsorbents. The synthesis reactions for both adsorbents are presented in eqn (1) and (2) below.1
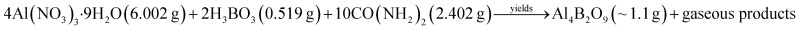
2
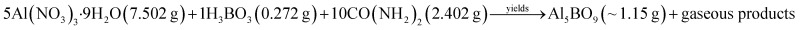


The synthesis procedure was identical for both adsorbents. The starting reagents were accurately weighed according to the calculated stoichiometric composition before synthesis. After weighing, the starting material reagents were transferred into a porcelain crucible and homogenized before thermal treatment. Subsequently, 50 mL of distilled water was added to dissolve the mixtures. The resulting solutions were placed on a hot plate set to 100 °C to evaporate the water. After complete evaporation, the remaining solid mixtures were thoroughly homogenized. These mixtures were then transferred to a furnace and maintained at 500 °C for 15 minutes. After cooling, the obtained powder was ground thoroughly to ensure compositional homogeneity. The homogenized powder mixtures were then separately placed into aluminum oxide boats. The homogenized powder was transferred to an alumina boat and calcined at 850 °C for 6 h. The furnace was operated under atmospheric conditions.

### Batch adsorption experiments

MeB removal experiments were performed in batch mode under controlled solution conditions. The influence of key operating variables, namely pH, adsorbent amount, contact time, initial MeB concentration, and temperature, was evaluated to determine their effects on adsorption performance. The pH range selected for the experiments was 3–9, while temperature studies were carried out within the range of 25 to 55 °C. The effect of the initial dye concentration was evaluated at concentrations ranging from 25 to 200 ppm. To determine the influence of adsorbent dosage, a series of adsorbent dosages between 0.01 and 0.09 g were selected. The pH of the solutions was adjusted using 0.1 M HCl and 0.1 M NaOH. The pH meter was calibrated with appropriate buffer solutions prior to measurements. Batch adsorption experiments were performed using 30 mL of MeB solution in 50 mL containers at a shaking speed of 150 rpm. After adsorption, the suspensions were filtered using filter paper, and the filtrates were diluted to a final volume of 50 mL with distilled water prior to UV-Vis analysis. The measured concentrations were corrected for dilution before calculating adsorption capacity. The amount of dye adsorbed was determined using a UV-Vis spectrophotometer at the maximum wavelength of the dye. In the equilibrium studies, the amount of MeB adsorbed onto Al_4_B_2_O_9_ and Al_5_BO_9_ was calculated using a mass balance approach. The equilibrium adsorption capacity of the dye was determined using eqn (3).3
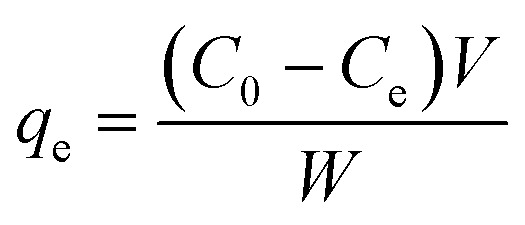


*C*_0_ represents the initial concentration of MeB, *C*_e_ denotes the equilibrium MeB concentration after dilution correction, *W* (g) corresponds to the amount of Al_4_B_2_O_9_ and Al_5_BO_9_ used as adsorbents, and *V* (L) is the volume of the solution.^1^ Subsequently, kinetic and isotherm studies were conducted under selected fixed experimental conditions, as specified in the corresponding figure captions. The kinetic experiments were performed by varying the contact time between 30 and 120 minutes, whereas the adsorption isotherms were evaluated at initial dye concentrations ranging from 25 to 200 mg L^−1^.

## Results & discussion

### N_2_ adsorption–desorption analysis

**Fig. 1:**
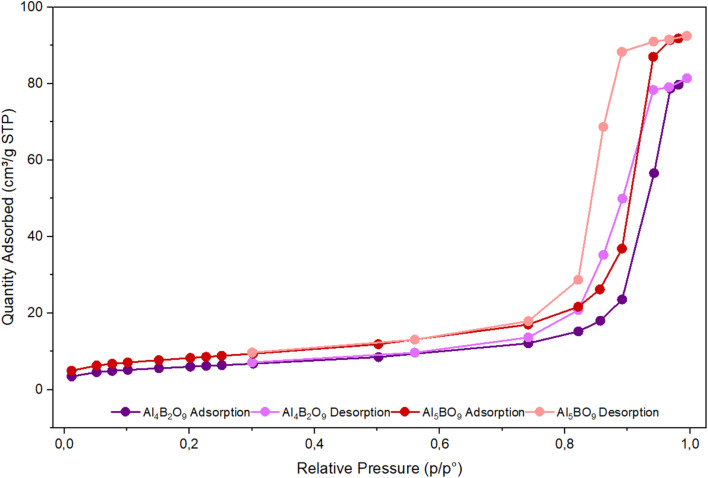
N_2_ adsorption–desorption isotherms of Al_4_B_2_O_9_ and Al_5_BO_9_.

The Brunauer–Emmett–Teller (BET) method is a widely used technique for determining the specific surface area of porous materials, including solids. The specific surface area of solid adsorbents is often associated with their adsorption performance, as it may be related to the number of active sites available for pollutant binding.^15^ Surface area becomes particularly significant when working with materials possessing a porous structure. The specific surface area and pore volume of the synthesized materials were determined from N_2_ adsorption–desorption isotherms at 77 K. Fig. 1 presents the comparative BET adsorption isotherms of Al_4_B_2_O_9_ and Al_5_BO_9_.

The results provide information regarding the surface area and pore structure of the porous materials. Fig. 1 indicates a positive correlation between the increase in relative pressure (*P*/*P*_0_) and the amount of gas adsorbed. Furthermore, the isotherm curve exhibits a pronounced upward trend, particularly as the relative pressure approaches unity. The pore size distribution was calculated using the Barrett–Joyner–Halenda (BJH) model.^16^ The BET analysis results for Al_4_B_2_O_9_ and Al_5_BO_9_ are presented in Table 2.

**Table 2:** Textural properties of Al_4_B_2_O_9_ and Al_5_BO_9_ obtained from N_2_ adsorption–desorption analysis

Materials	Single point (m^2^ g^−1^)	BET (m^2^ g^−1^)	*t*-Plot micropore (m^2^ g^−1^)	*t*-Plot external (m^2^ g^−1^)	BJH adsorption (m^2^ g^−1^)	Single point adsorption (cm^3^ g^−1^)	*t*-Plot micropore (cm^3^ g^−1^)	BJH adsorption (cm^3^ g^−1^)
Al_4_B_2_O_9_	20.966	21.473	2.653	18.821	23.344	0.126	0.001	0.125
Al_5_BO_9_	28.893	29.599	3.114	26.485	33.286	0.143	0.001	0.142

Table 2 shows that the specific surface areas of Al_4_B_2_O_9_ and Al_5_BO_9_ are 21.473 m^2^ g^−1^ and 29.599 m^2^ g^−1^, respectively, while their pore volumes are 0.125 cm^3^ g^−1^ and 0.142 cm^3^ g^−1^, respectively. Compared with Al_4_B_2_O_9_, Al_5_BO_9_ exhibited higher BET surface area and pore volume, suggesting greater surface accessibility and more pronounced pore development. These textural properties may partially contribute to the higher adsorption capacity of Al_5_BO_9_. However, aqueous dye adsorption is not governed solely by BET surface area; surface charge, solution pH, surface active sites, and specific adsorbent–adsorbate interactions may also play important roles in MeB adsorption.

### FT-IR analysis of synthesized aluminum borates

**Fig. 2:**
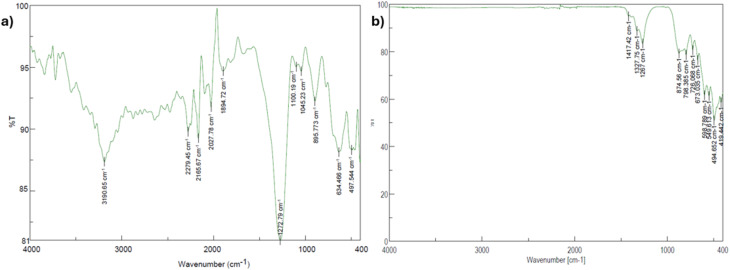
FT-IR spectra of combustion-synthesized aluminum borates: (a) Al_4_B_2_O_9_ (b) Al_5_BO_9_.

The characteristic vibrational bands of the synthesized aluminum borate compounds were evaluated using FT-IR spectroscopy. The FT-IR spectra of the Al_4_B_2_O_9_ and Al_5_BO_9_ materials are presented in Fig. 2. FT-IR spectra in the range of 4000–400 cm^−1^ were recorded at room temperature using a JASCO FT/IR-4700 Type A spectrometer. The FT-IR spectra exhibited several characteristic absorption bands corresponding to the vibrational modes of B–O and Al–O bonds. In the Al_4_B_2_O_9_ material, the band observed at 3190 cm^−1^ is attributed to O–H stretching vibrations, indicating the presence of hydroxyl groups within the sample. This peak was not observed in the Al_5_BO_9_ material; instead, its main absorption bands were found to begin at approximately ∼1420 cm^−1^. For both materials, the bands detected in the range of ∼1330–1270 cm^−1^ are considered to correspond to the asymmetric stretching vibrations of B–O–B linkages. The bands observed between ∼1100 and 1000 cm^−1^ are associated with B–O stretching vibrations of BO_3_ structural units. The bands in the ∼700–600 cm^−1^ region suggest the presence of Al–O vibrations, while those at ∼500 cm^−1^ and below are attributed to AlO_6_ units.^17–19^

### Morphological analysis by SEM

**Fig. 3:**
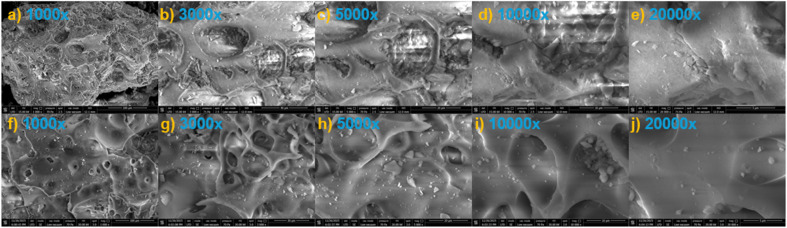
SEM micrographs of Al_4_B_2_O_9_ and Al_5_BO_9_ at different magnifications: (a–e) Al_4_B_2_O_9_ and (f–j) Al_5_BO_9_.

The surface morphology of the Al_4_B_2_O_9_ and Al_5_BO_9_ materials was examined using scanning electron microscopy (SEM) at magnifications ranging from 1000× to 200 00×. The SEM images of Al_4_B_2_O_9_ and Al_5_BO_9_ are presented in Fig. 3. SEM observations provide useful morphological information for adsorption studies by revealing surface roughness, irregular particles, pore-like regions and microstructural heterogeneity. At magnifications of 1000× and 2000×, Al_4_B_2_O_9_ exhibits a highly rough surface with visible void like regions and channel like features. In contrast, Al_5_BO_9_ appears to display a more homogeneous structure at lower magnifications. At 3000× and 5000× magnifications, Al_4_B_2_O_9_ shows ring-like and layered formations with partially melted and cracked regions. These structures may be attributed to irregular sintering processes or phase separation. At higher magnifications (100 00× and 200 00×), the surface topography of Al_4_B_2_O_9_ is observed to consist of more compact areas along with micro porous regions. For Al_5_BO_9_, the images at 100 00× and 200 00× magnifications suggest fewer visible surface voids and smoother grain surfaces. Overall, Al_5_BO_9_ appears to possess a more homogeneous microstructure in the SEM images. However, its higher BET surface area and pore volume suggest that smaller-scale porosity may be more developed than can be directly observed from SEM micrographs.

### Phase structure and crystallinity by XRD

**Fig. 4:**
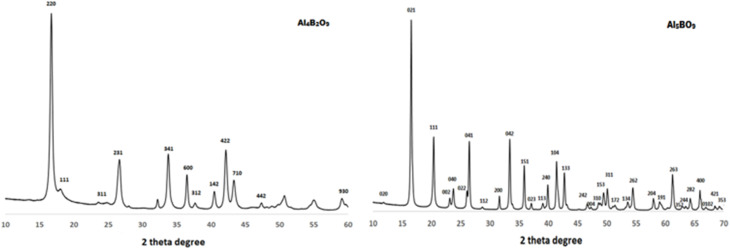
XRD patterns of combustion-synthesized Al_4_B_2_O_9_ and Al_5_BO_9_.

The crystalline phase formation and structural characteristics of the synthesized adsorbents were evaluated by X-ray diffraction (XRD) analysis. The XRD patterns of Al_4_B_2_O_9_ and Al_5_BO_9_ prepared *via* the combustion method are presented in Fig. 4. When the diffraction peaks obtained for both adsorbents Al_4_B_2_O_9_ and Al_5_BO_9_ are compared with standard card no: 00-029-0010 and 01-077-0395, respectively, they are found to be consistent. Furthermore, previous studies have reported that Al_4_B_2_O_9_ has orthorhombic unit cells with values of *a* = 14.746 Å, *b* = 15.268 and *c* = 5.557 Å. In addition, it has been reported that the other adsorbent Al_5_BO_9_ also has orthorhombic unit cells with values of *a* = 5.682 Å, *b* = 14.973 and *c* = 7.692 Å.^19–22^

### Thermal stability of Al_4_B_2_O_9_ and Al_5_BO_9_

**Fig. 5:**
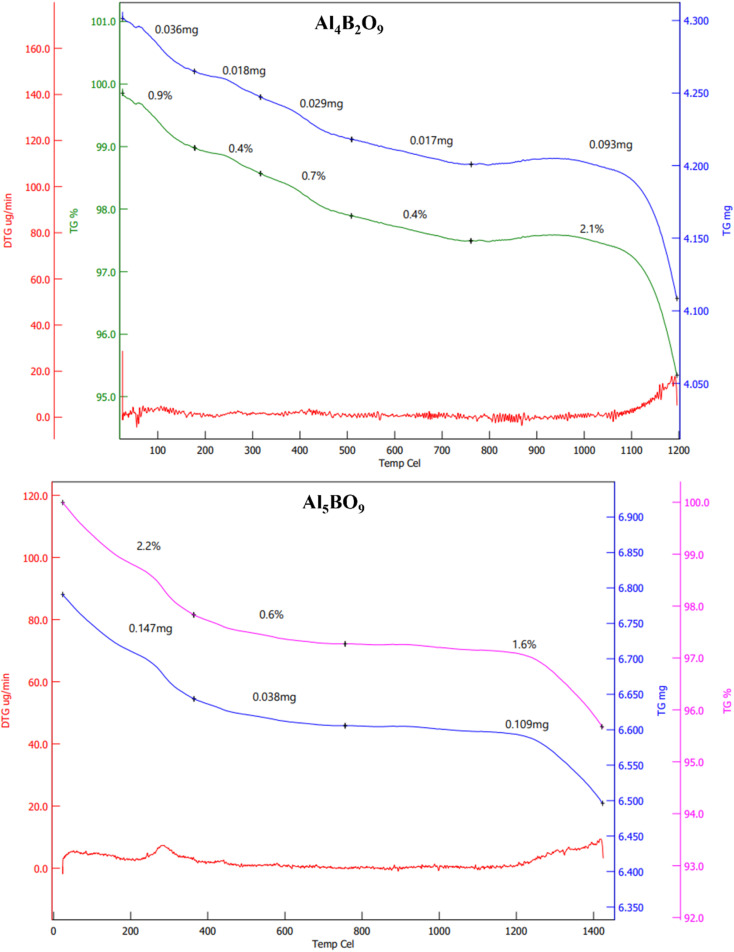
TG/DTG curves of Al_4_B_2_O_9_ and Al_5_BO_9_.

Fig. 5 presents the TG/DTG curves of the Al_4_B_2_O_9_ and Al_5_BO_9_ materials. Thermal analysis was carried out over the temperature range of 25 to 1400 °C. For the Al_4_B_2_O_9_ material, a gradual but low-rate mass loss is observed. The most pronounced mass loss occurs between 1000 °C and 1200 °C. The total mass loss was determined to be approximately 4%. Similarly, the Al_5_BO_9_ material exhibits a controlled and relatively low mass loss. However, the total mass loss for Al_5_BO_9_ is approximately 6%. The sharpest decrease in mass is observed in the temperature range of 1200 to 1400 °C. The results indicate that both materials demonstrate high thermal stability up to 1000 °C. The more significant mass losses observed above 1000 °C may be associated with structural rearrangements within the borate framework, possible structural rearrangements, phase related changes or dehydroxylation and dehydration processes.

### Zeta potential and effect of initial solution pH

**Fig. 6:**
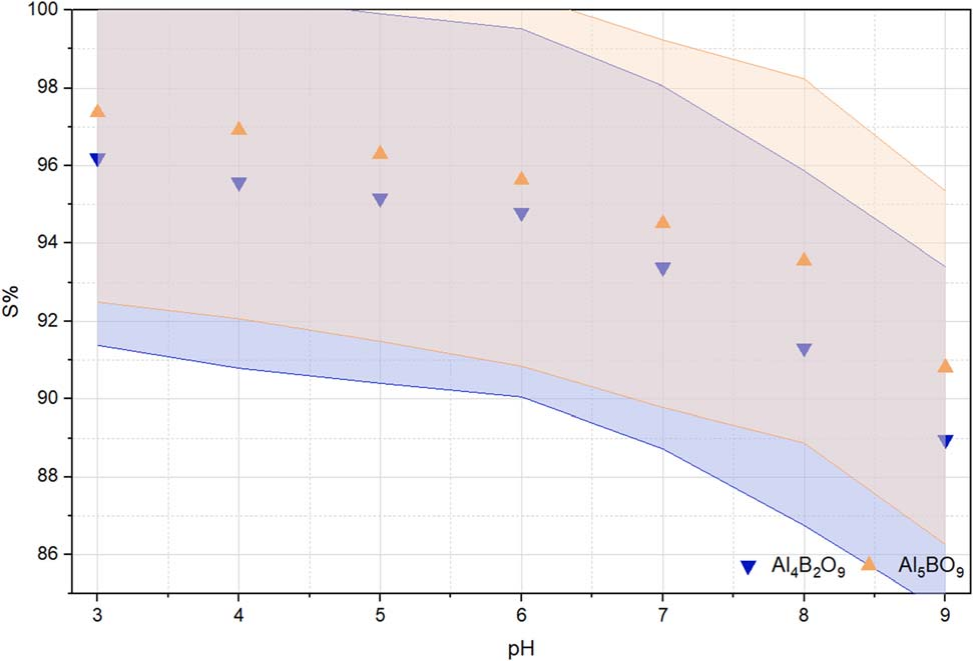
Effect of initial solution pH on MeB removal by Al_4_B_2_O_9_ and Al_5_BO_9_ (conditions: concentration: 50 mg L^−1^, dosage: 0.05 g, temperature: 25 °C, time: 60 min, pH: 3–9).

In aqueous media, solution pH strongly affects the electrical double layer and therefore the zeta potential of solid particles. Zeta potential measurements provide information on surface-charge variation with pH and help interpret the electrostatic contribution to dye adsorption.^23^ In dye adsorption studies, it has been reported that the surface charge, as interpreted from zeta potential results, can significantly influence the adsorption performance of a material through its interaction with dye molecules.^24^ Zeta potential measurements were performed by dispersing 50 mg of the sample in 10 mL of ultrapure water. The suspension was homogenized using a vortex mixer and subsequently centrifuged at 1000 rpm. The resulting supernatant was then transferred into an electrode-equipped measurement cell, and the zeta potential was measured using the instrument. An examination of the zeta potential and electrophoretic mobility results for Al_4_B_2_O_9_ and Al_5_BO_9_ shows that the zeta potential values were −11.5 mV and −50.7 mV, respectively. The corresponding electrophoretic mobility values were determined as −0.000089 cm^2^ V^−1^ s^−1^ and −0.000391 cm^2^ V^−1^ s^−1^. These results indicate that Al_4_B_2_O_9_ exhibits weak electrostatic repulsion and a higher tendency toward aggregation, whereas Al_5_BO_9_ demonstrates strong electrostatic repulsion. Solution pH is a key variable in adsorption because it modifies adsorbent surface charge, functional-group protonation, and the interaction strength between adsorbent and adsorbate.^25,26^ The adsorption capability may depend on the electrostatic interactions between MeB molecules and the adsorbent surface. The variation in the percentage of dye adsorbed at equilibrium as a function of solution pH is shown in Fig. 6. Maximum dye adsorption for both adsorbents was observed at pH 3. The removal efficiency of MeB decreased as the pH increased from 3 to 9. MeB, an anionic dye molecule, carries a negative charge due to the presence of sulfonate groups (SO_3_^−^), which ionize in aqueous solution. Under acidic conditions, protonation of Al–O and B–O related surface hydroxyl groups may decrease the negative charge density of the borate surface, which could reduce electrostatic repulsion and favor the adsorption of anionic MeB species. Therefore, MeB can retain its anionic character even under acidic conditions due to its sulfonate groups. Surface Al-based sites may interact with the negatively charged sulfonate groups of MeB, thereby contributing to adsorption under acidic conditions. In basic environments, sulfonate groups remain in their anionic –SO_3_^−^ form and thus retain their negative charge. Under alkaline conditions, the adsorption of hydroxide ions may increase the negative surface character of aluminum borate compounds. Consequently, electrostatic repulsion occurs between the negatively charged dye molecules and the negatively charged adsorbent surface, resulting in reduced adsorption compared with acidic conditions. Similar findings have been reported in the literature. Zhao *et al.* (2012) observed a decrease in adsorption capacity with increasing pH in the removal of Acid Red 18 dye.^27^ Likewise, Bayram *et al.* (2026) reported a significant decrease in adsorption percentage with increasing pH during the adsorption of the anionic dye alizarin yellow GG.^28^

### Effect of adsorbent dosage on MeB removal

**Fig. 7:**
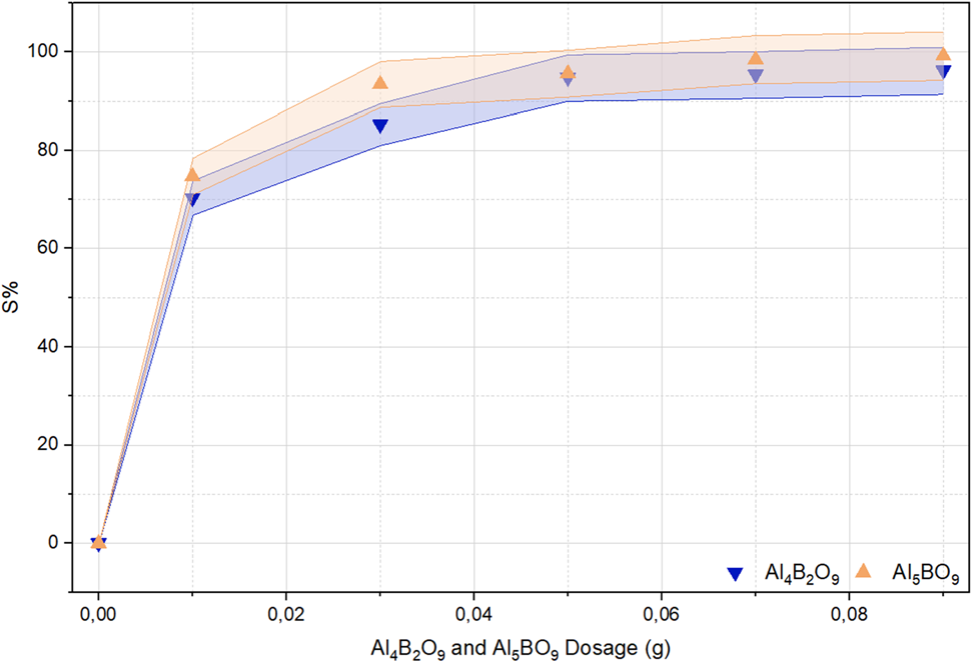
Effect of adsorbent dosage on MeB removal by Al_4_B_2_O_9_ and Al_5_BO_9_ (conditions: concentration: 50 mg L^−1^, dosage: 0.01 g, 0.03 g, 0.05 g, 0.07 g, 0.09 g, temperature: 25 °C, time: 60 min, pH: natural).

Adsorbent dosage, evaluated under various adsorption conditions, is considered a significant parameter in determining the practical applicability of an adsorbent.^29^ The effect of varying adsorbent dosage on MeB removal using Al_4_B_2_O_9_ and Al_5_BO_9_ is presented in Fig. 7. It is well established that the efficiency of dye adsorption is strongly influenced by the amount of adsorbent employed. Increasing the adsorbent dosage is generally associated with an enhancement in dye removal due to the greater availability of active adsorption sites.^30,31^ In the present study, an increase in the dosage of both Al_4_B_2_O_9_ and Al_5_BO_9_ led to a corresponding increase in dye removal efficiency. When the adsorbent dosage was increased from 0.01 to 0.09 g, the removal efficiency for Al_4_B_2_O_9_ increased from 70.34 to 96.24%. Similarly, for Al_5_BO_9_, the removal efficiency increased from 74.72 to 99.21%. These increases were observed to stabilize beyond a certain adsorbent amount.^32^ Accordingly, for both materials, the removal efficiency remained relatively constant at dosages above 0.05 g. Since the removal efficiency showed only a slight increase above 0.05 g, this dosage was selected as the practical optimum for subsequent experiments.

### Effect of contact time and adsorption kinetics

**Fig. 8:**
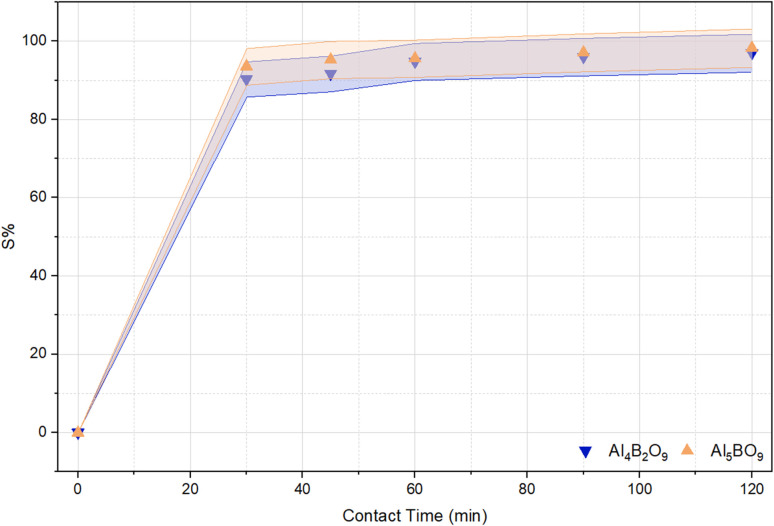
Effect of contact time on MeB removal by Al_4_B_2_O_9_ and Al_5_BO_9_ (conditions: concentration: 50 mg L^−1^, dosage: 0.05 g, temperature: 25 °C, time: 30 min, 45 min, 60 min, 90 min, 120 min, pH: natural).

Contact time is an important operational variable because it determines the duration of interaction between dye molecules and active adsorption sites. As the dye adsorbate approaches the active sites of the adsorbent, adsorption efficiency is expected to increase.^33^ The effect of contact time on the sorption efficiency (%*S*) of MeB removal using Al_4_B_2_O_9_ and Al_5_BO_9_ is presented in Fig. 8. Contact times were systematically investigated within the range of 30 to 120 minutes. The adsorption efficiencies of Al_4_B_2_O_9_ and Al_5_BO_9_ at 30 minutes were determined to be 90.30% and 93.55%, respectively. At 120 minutes, these values increased to 97.03% and 98.31%, respectively. The adsorption efficiency increased with contact time until equilibrium was approached.^34^ At the initial stage, the adsorption process proceeds rapidly due to the availability of vacant active sites on the surface. However, both adsorbents were observed to reach saturation within approximately 60 minutes. Beyond 60 minutes, no significant change in adsorption percentage was detected. Over time, as the adsorption sites on the surface of the aluminum borate compounds become occupied, the adsorption rate decreases. Eventually, the rates of adsorption and desorption reach equilibrium at the final equilibrium point. Furthermore, the kinetic parameter results are presented in Table 3.

**Table 3:** Kinetic parameters for MeB adsorption onto Al_4_B_2_O_9_ and Al_5_BO_9_

Sorbents	Model	*q*_e_ (mg g^−1^)	*k*	*C* (mg g^−1^)	*R* ^2^
Al_4_B_2_O_9_	PFO	29.109	0.033 min^−1^		0.968
	PSO	29.927	0.010 g mg^−1^ min^−1^		0.999
	Intraparticle diffusion		0.380 mg g^−1^ min^−1/2^	25.124	0.928
Al_5_BO_9_	PFO	29.193	0.051 min^−1^		0.931
	PSO	29.624	0.020 g mg^−1^ min^−1^		0.999
	Intraparticle diffusion		0.202 mg g^−1^ min^−1/2^	27.101	0.920

**Fig. 9:**
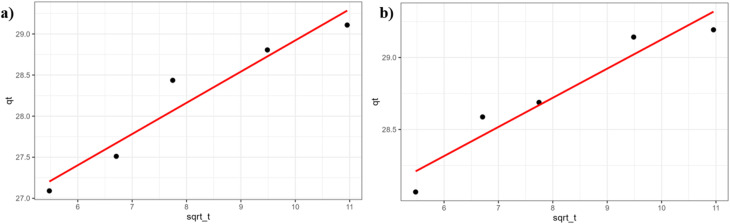
Intraparticle diffusion plots for MeB adsorption. (a) Al_4_B_2_O_9_ (b) Al_5_BO_9_.

Adsorption kinetics plays a crucial role in understanding dye adsorption processes. The relationship between contact time and adsorption capacity is considered instrumental in determining the appropriate kinetic model and its associated parameters.^35^ The adsorption rate is governed by adsorption kinetics, which in turn provides insight into the underlying adsorption mechanism.^36^ The adsorption process of MeB dye was investigated using the pseudo-first-order (PFO), pseudo-second-order (PSO), and intraparticle diffusion kinetic models. Among the kinetic models tested, the PSO model yielded the most suitable description of MeB adsorption on both Al_4_B_2_O_9_ and Al_5_BO_9_, with *R*^2^ values approaching unity. The kinetic parameters were calculated using adsorption capacity values obtained from the mass balance equation based on the initial adsorption volume. The better agreement with the PSO model suggests that the adsorption rate is closely associated with the availability of surface active sites. However, this result alone does not conclusively confirm chemisorption. The intraparticle diffusion model plot is shown in Fig. 9.

### Effect of temperature and adsorption thermodynamics

**Fig. 10:**
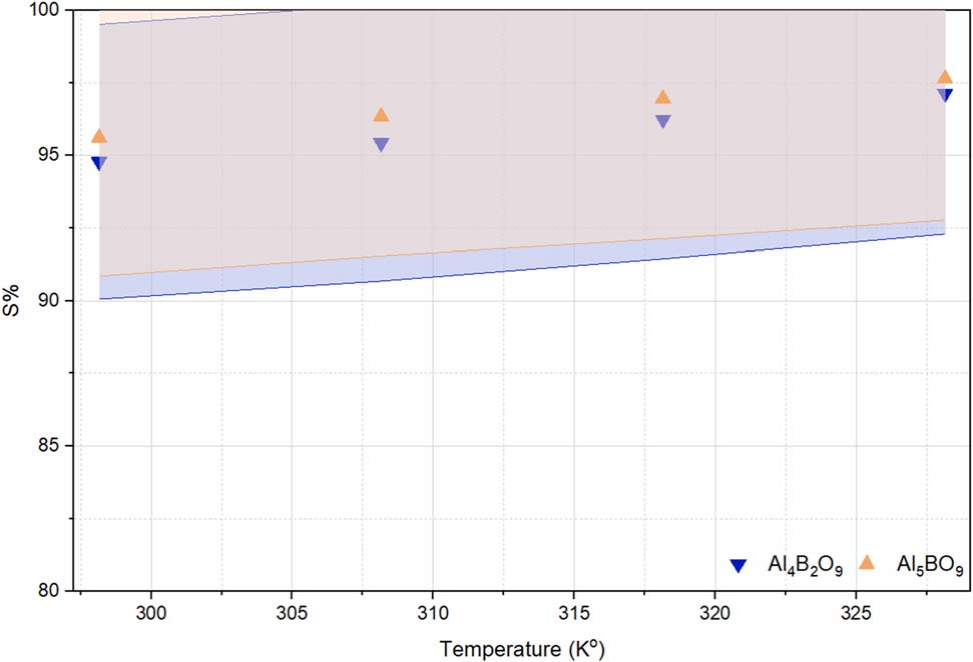
Effect of temperature on MeB removal by Al_4_B_2_O_9_ and Al_5_BO_9_ (conditions: concentration: 50 mg L^−1^, dosage: 0.05 g, temperature: 25 °C, 35 °C, 45 °C, 55 °C, time: 60 min, pH: natural).

Since adsorption equilibrium can vary with temperature, thermodynamic parameters were evaluated to clarify the thermal nature of MeB removal. In this study, temperatures of 25 °C, 35 °C, 45 °C, and 55 °C were selected to evaluate the effect of temperature, while the remaining parameters were kept constant. The influence of temperature variation on the sorption efficiency of MeB removal using Al_4_B_2_O_9_ and Al_5_BO_9_ is presented in Fig. 10. The removal efficiencies of Al_4_B_2_O_9_ and Al_5_BO_9_ at 25 °C were determined to be 94.79% and 95.63%, respectively. At 55 °C, these values increased to 97.14% and 97.65%, respectively. It is well established that temperature variations affect both the thermodynamics and kinetics of adsorption processes. The increase in MeB removal efficiency with temperature suggests that adsorption onto both aluminum borates is favored at higher temperatures.^37^ The results demonstrate that MeB removal efficiency increases with rising temperature, indicating that the adsorption process is favored at higher temperatures.^38^

Thermodynamic studies were conducted to investigate the effect of temperature on adsorption capacity. The calculated Δ*G*°, Δ*H*°, and Δ*S*° values for MeB adsorption are summarized in Table 4. The equilibrium constant K was calculated in dimensionless form before estimating Δ*G*°, Δ*H*°, and Δ*S*°, in order to avoid the direct use of dimensional adsorption coefficients in thermodynamic calculations. The Gibbs free energy of adsorption was calculated using eqn (4) provided below.

4 Δ*G*° = Δ*H*° − *T*Δ*S*°

Accordingly, the thermochemical parameters Δ*H*° and Δ*S*° were calculated by applying the van't Hoff equation (eqn (5)).5
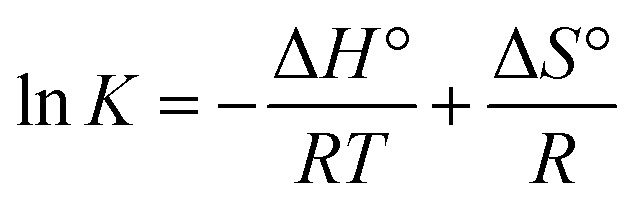


**Table 4:** Thermodynamic parameters for MeB adsorption onto Al_4_B_2_O_9_ and Al_5_BO_9_

	*T* (K)	298.150	308.150	318.150	328.150
Al_4_B_2_O_9_	Δ*G*° (kJ mol^−1^)	−7.091	−7.893	−8.695	−9.498
	Δ*S*° (J mol^−1^ K^−1^)	80.222			
	Δ*H*° (kJ mol^−1^)	16.827			
Al_5_BO_9_	Δ*G*° (kJ mol^−1^)	−7.598	−8.428	−9.257	−10.087
	Δ*S*° (J mol^−1^ K^−1^)	82.940			
	Δ*H*° (kJ mol^−1^)	17.130			

*R* represents the universal gas constant. The enthalpy change (Δ*H*°) and entropy change (Δ*S*°) values were determined from the slope and intercept of the linear plot of ln *K versus* 1/*T*, in accordance with the van't Hoff approach.^39^ The standard enthalpy change (Δ*H*°) was determined to be 16.827 kJ mol^−1^ for Al_4_B_2_O_9_ and 17.130 kJ mol^−1^ for Al_5_BO_9_. Positive Δ*H*° values indicate that MeB adsorption increases with heat input and that the process proceeds *via* an endothermic pathway. The Δ*G*° values became more negative with increasing temperature, changing from −7.091 to −9.498 kJ mol^−1^ for Al_4_B_2_O_9_ and from −7.598 to −10.087 kJ mol^−1^ for Al_5_BO_9_ as the temperature increased from 298 to 328 K. The Δ*G*° results confirm that the adsorption of MeB is both favorable and spontaneous. The positive value of Δ*S*° suggests a change in the degree of disorder at the adsorbent/solution interface during adsorption, suggesting increased randomness at the solid solution interface during MeB adsorption.^40^

**Fig. 11:**
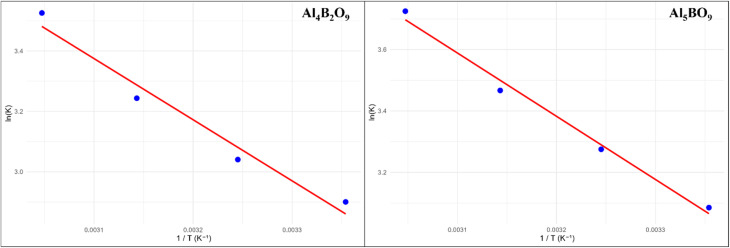
Van't Hoff plots for MeB adsorption onto Al_4_B_2_O_9_ and Al_5_BO_9_ (ln *K* − 1/*T*).

Fig. 11 presents the variation of ln *K* with respect to 1/*T* for the Al_4_B_2_O_9_ and Al_5_BO_9_ materials. The linear and negative trend obtained indicates that the systems follow Van't Hoff behavior. The negative slope of the ln *K versus* 1/*T* plot is consistent with the positive Δ*H*° values, supporting the endothermic nature of MeB adsorption.

### Effect of initial MeB concentration and adsorption isotherms

The initial dye concentration is a critical parameter in adsorption studies, as it directly influences the driving force for mass transfer.^41^ The initial concentration of MeB is considered to be closely associated with the adsorption capacity. In this study, MeB concentrations ranging from 25 to 200 mg L^−1^ were investigated. The other experimental conditions were kept constant at an adsorbent dosage of 0.05 g, a temperature of 25 °C and a contact time of 60 minutes. As the MeB concentration increases while the adsorbent dosage remains constant, the adsorbent surface may approach saturation. This behavior can be attributed to the limited number of active adsorption sites available on Al_4_B_2_O_9_ and Al_5_BO_9_. Consequently, different maximum adsorption values are obtained for varying initial concentrations at a fixed adsorbent dosage. Adsorption isotherm parameters are widely used to describe how adsorbate molecules interact with adsorbent surface sites and how equilibrium is established. These parameters provide insight into the adsorption capacity of the adsorbent, the nature of the adsorption process, and its overall efficiency. Langmuir,^42^ Freundlich,^43^ Scatchard,^44^ and Temkin^45^ models were applied to interpret the equilibrium behavior of MeB adsorption and to assess the adsorption capacity of the synthesized borates, as presented in Table 5.

Isotherm model parameters for MeB adsorption onto Al_4_B_2_O_9_ and Al_5_BO_9_

**Table:** 

Isotherm	Isotherm parameters linear form									
Langmuir (1/*q*_e_*vs.* 1/*C*_e_)	*q*_max_	*K*_L_ (L mg^−1^)	*R* ^2^							
Al_4_B_2_O_9_	70.547	1.87	0.966	*C*_0_	25	50	75	100	150	200
				*R*_L_	0.0209	0.0106	0.0071	0.0053	0.0036	0.0027
Al_5_BO_9_	105.785	2.5	0.965	*C*_0_	25	50	75	100	150	200
				*R*_L_	0.0158	0.0080	0.0053	0.0040	0.0027	0.0020

**Table:** 

Isotherm	Isotherm parameters linear form		
Freundlich (log *C*_e_*vs.* log *q*_e_)	*K*_f_	1/*n*	*R* ^2^
Al_4_B_2_O_9_	21.146	0.488	0.986
Al_5_BO_9_	27.072	0.705	0.988

**Table:** 

Isotherm	Isotherm parameters linear form		
Scatchard (*q*_e_*vs. q*_e/_*C*_e_)	*Q*_s_	*K*_b_	*R* ^2^
Al_4_B_2_O_9_	98.903	0.268	0.680
Al_5_BO_9_	183.69	0.178	0.657

**Table:** 

Isotherm	Isotherm parameters linear form		
Temkin (ln *C*_e_*vs. q*_e_)	*B*_t_	*K*_t_	*R* ^2^
Al_4_B_2_O_9_	20.758	2.78	0.885
Al_5_BO_9_	32.899	2.63	0.895

Adsorption isotherm models, such as the Langmuir model, are frequently employed to describe complex adsorption behavior. The Langmuir isotherm assumes that adsorption occurs as a monolayer on a homogeneous surface with identical binding sites and without interaction between adsorbed molecules. In this model, *q*_max_ represents the maximum adsorption capacity, whereas *K*_L_ denotes the Langmuir adsorption equilibrium constant, which is commonly associated with adsorbate and adsorbent affinity.^42,46^ The higher *K*_L_ value obtained for Al_5_BO_9_ indicates a stronger binding affinity and higher interaction energy. The calculated separation factor (*R*_L_) values were found to be in the range of (Al_4_B_2_O_9_: 0.0027–0.0209, Al_5_BO_9_: 0.0020–0.0158) indicating highly favorable adsorption behavior. The *R*_L_ values approaching zero suggest a strong affinity between the adsorbate and the adsorbent surface, supporting the favorable nature of adsorption within the Langmuir framework. The suitability of each isotherm model was assessed by comparing the corresponding *R*^2^ values with the experimental equilibrium data. The model with a regression coefficient closest to unity is considered to provide the best fit to the adsorption process. For both adsorbents, the adsorption data were found to be more consistent with the Freundlich isotherm model (*R*^2^ = 0.986 and 0.988). According to the Freundlich model, the *K*_F_ constants were calculated as 21.146 for Al_4_B_2_O_9_ and 27.072 for Al_5_BO_9_. This behavior indicates that MeB removal is likely associated with heterogeneous adsorption sites and may involve multilayer adsorption contributions. Although the Freundlich model provided the best fit, the Langmuir model was also used to estimate the maximum monolayer adsorption capacities, which were determined as 70.547 mg g^−1^ for Al_4_B_2_O_9_ and 105.785 mg g^−1^ for Al_5_BO_9_. The Scatchard analysis yielded relatively low correlation coefficients (*R*^2^ ≈ 0.65–0.68) for both adsorbents, indicating that the adsorption process cannot be adequately described by a single class of homogeneous binding sites. Although the higher *Q*_s_ value obtained for Al_5_BO_9_ suggests a greater theoretical adsorption capacity, the poor fit implies that the model is not suitable for this system. Overall, the Scatchard model appears insufficient to accurately represent the adsorption mechanism. Similar studies on MeB removal reported in the literature are summarized in Table 6.

Comparison of MeB adsorption capacities reported for different adsorbents

**Table:** 

Adsorbent	Adsorption capacity (mg g^−1^)	References
MGLfsB	11.148	47
nM-MGLfsB	13.089	47
Magnetic Ni_0.1_Mg_0.7_Co_0.2_Fe_2_O_4_	2784.100	48
Activated carbon	53.000	48
Novel CaCO_3/_chitin aerogel	266.400	48
Na_2_Al_2_B_2_O_7_	2000.000	49

**Table:** 

Adsorbent	Adsorption capacity (mg g^−1^)		References
	Langmuir	Freundlich (*K*_F_) constant	
Al_5_BO_9_	105.785 mg g^−1^	27.072	This study
Al_4_B_2_O_9_	70.547 mg g^−1^	21.146	This study

Various methods and material systems have been developed in the literature for dye removal. Hybrid flocculation-photocatalysis systems have been reported to achieve high removal efficiencies,^50^ while dual-functional materials such as Fe@ZIF-8 enable effective treatment by combining adsorption with advanced oxidation processes.^51^ In addition, Z-scheme photocatalysts have demonstrated rapid degradation under visible light irradiation.^52^ In contrast, the present study focuses on the synthesis of aluminum borates (Al_4_B_2_O_9_ and Al_5_BO_9_), offering a different adsorption-based approach.

### Proposed adsorption mechanism

**Fig. 12:**
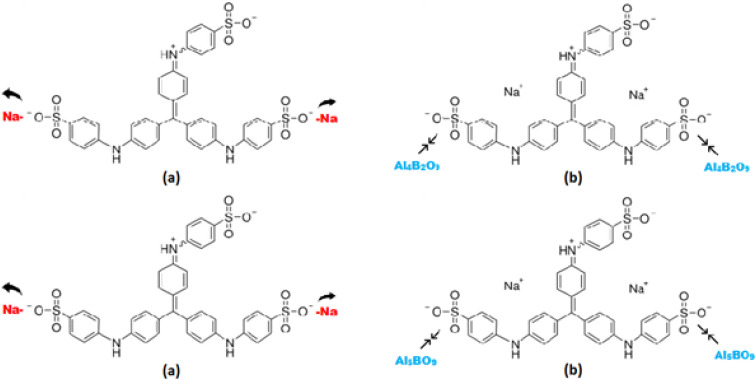
Proposed adsorption mechanism of MeB onto Al_4_B_2_O_9_ and Al_5_BO_9_: (a) and (b) represent possible adsorption pathways.

Al_4_B_2_O_9_ and Al_5_BO_9_ are oxygen-rich aluminum borate structures, which may provide surface oxygen-containing sites involved in MeB adsorption. The measured negative zeta potential values support that the borate surfaces possess negatively charged sites under the selected measurement conditions. The ionization behavior of MeB, an anionic dye, in aqueous medium is illustrated in Fig. 12a. In aqueous solution, MeB exists predominantly as an anionic dye due to the dissociation of sulfonate groups. Under acidic conditions, partial protonation of surface hydroxyl sites on aluminum borates may reduce electrostatic repulsion and promote interactions between the adsorbent surface and the anionic sulfonate groups of MeB. In addition to electrostatic interactions, possible interactions with Al-based surface sites and pore filling effects may contribute to the overall adsorption process. The proposed adsorption mechanism between MeB and the aluminum borate surfaces is schematically presented in Fig. 12a and b.

## Conclusion

In recent years, various materials have been investigated for the adsorption of MeB dye from wastewater, and researchers have extensively examined the effectiveness of different adsorbents for this application. In the present study, Al_4_B_2_O_9_ and Al_5_BO_9_ were synthesized and characterized. Subsequent systematic adsorption experiments revealed that maximum MeB adsorption occurred at pH 3, while removal efficiency decreased progressively toward pH 9. Under acidic conditions, partial protonation of surface hydroxyl sites may reduce electrostatic repulsion and facilitate the adsorption of anionic MeB species onto aluminum borate surfaces. Although sulfonate groups remain negatively charged due to their strong acidity, they may interact with surface Al-based sites, thereby contributing to MeB adsorption. In alkaline conditions, both the dye molecules and the aluminum borate surface carry negative charges, leading to electrostatic repulsion and reduced adsorption. The removal efficiency of MeB increased with increasing adsorbent dosage, reaching a maximum of 96.24% for Al_4_B_2_O_9_ and 99.21% for Al_5_BO_9_. However, no substantial improvement in removal efficiency was observed beyond 0.05 g, indicating that this dosage represented a practical optimum under the studied conditions. Equilibrium was reached within approximately 60 min, with rapid removal at the initial stage followed by a slower adsorption rate as the available surface sites became occupied. The PSO model most suitably described the kinetic behavior of MeB adsorption on both borates, suggesting that the rate was mainly governed by the accessibility of active adsorption sites. An increase in temperature resulted in a corresponding increase in the amount of MeB adsorbed by the adsorbents. This finding indicates that MeB adsorption onto both borates proceeds as an endothermic adsorption process (Δ*H* = 16.827 kJ mol^−1^ for Al_4_B_2_O_9_; 17.130 kJ mol^−1^ for Al_5_BO_9_). The adsorption data were more consistent with the Freundlich isotherm model (*R*^2^ = 0.986 and 0.988), suggesting adsorption on energetically heterogeneous surface sites with a possible contribution from multilayer adsorption. A key limitation of this study is that it was conducted using a single component dye system under controlled laboratory conditions, which may restrict its direct applicability to complex real wastewater matrices containing multiple competing contaminants. In addition, the reusability and regeneration performance of the adsorbents were not evaluated, statistical error analysis was not performed, and shorter contact times were not investigated, which remain critical factors for future studies in terms of large scale application and economic feasibility.

## Informed consent

All authors agree to this publication.

## Author contributions

U. Özkan: formal analysis, resources, methodology, investigation, writing – original draft, writing – review & editing, software, validation, visualization. O. Bayram: conceptualization, formal analysis, investigation, methodology, project administration, software, validation, visualization. E. Moral: conceptualization, data curation, investigation, validation, visualization, writing – original draft, writing – review & editing. İ. Pekgözlü: investigation, methodology, writing – original draft, writing – review & editing. F. Göde: data curation, conceptualization, formal analysis, supervision, writing – original draft, writing – review & editing.

## Conflicts of interest

The authors declare that they have no conflict of interest.

## Data Availability

Data will be available on reasonable request.
